# Pan-Cancer Upregulation of the *FOXM1* Transcription Factor

**DOI:** 10.3390/genes16010056

**Published:** 2025-01-06

**Authors:** Daniele Pozzobon, Arianna Bellezza, Federico M. Giorgi

**Affiliations:** 1 Department of Computer Science, Free University, 1081 HV Amsterdam, The Netherlands; d.pozzobon@student.vu.nl; 2 Department of Pharmacy and Biotechnology, University of Bologna, 40138 Bologna, Italy; arianna.bellezza@studio.unibo.it

**Keywords:** *FOXM1*, cancer, transcriptomics, RNA-Seq, scRNA-Seq

## Abstract

Background: The human *FOXM1* transcription factor controls cell cycle progression and genome stability, and it has been correlated to the onset and progression of many tumor types. Methods: In our study, we collected all recent sequence and quantitative transcriptomics data about *FOXM1*, testing its presence across vertebrate evolution and its upregulation in cancer, both in bulk tissue contexts (by comparing the TCGA tumor dataset and the GTEx normal tissue dataset) and in single-cell contexts. Results: *FOXM1* is significantly and consistently upregulated in all tested tumor types, as well as in tumor cells within a cancer microenvironment. Its upregulation reverberates in the upregulation of its target genes and can be used as a biomarker for poor cancer outcome in at least four tumor types. Conclusions: Despite its lack of cancer-related mutations and amplifications, the recurring upregulation of *FOXM1* in all tumors puts a focusing lens on this gene as a candidate pan-cancer master regulator.

## 1. Introduction

Cancer still constitutes a major global health challenge, with an estimated 9.7 million deaths annually and over 20 million new cases diagnosed each year [1]. While each cancer type possesses peculiar (epi)genetic and molecular features which lead to tumor initiation, progression and maintenance, all cancers ultimately leverage similar downstream molecular pathways, such as uncontrolled cell proliferation, deregulated metabolism, and immunoevasion [2]. Since the cause of cancer is eminently molecular, the current overarching direction of cancer research is aimed towards the identification of molecules (e.g., nucleotides, proteins, metabolites) which can be causally connected to tumor insurgence [3] and therefore used both as diagnostic biomarkers and potential pharmacological targets for therapy [4]. Our war against cancer therefore requires a complete knowledge of its molecular drivers. One such driver has been shown to be the *FOXM1* gene, which has previously been linked to cancer initiation and progression [5].

The *FOXM1* (Forkhead Box M1) gene in humans encodes for a transcription factor belonging to the larger family of Forkhead Box (FOX) proteins, which are evolutionarily conserved across a wide range of species [6]. The FOX proteins are known for their distinct Forkhead (FH) DNA-binding domain, which plays a pivotal role in regulating gene expression linked to cell proliferation, differentiation, development, and homeostasis in both normal and pathological conditions [7]. In humans, *FOXM1* is particularly significant for its critical role as a regulator of a plethora of genes responsible for cell proliferation, as well as the maintenance of genomic stability [8].

From a pathway perspective, *FOXM1* is the final effector of several signaling cascades associated with cell cycle progression, especially during the phase transitions [9]. *FOXM1* primarily functions during the G1/S and G2/M transitions of the cell cycle, where it orchestrates the transcription of genes required for DNA replication, mitosis, and cytokinesis [10].

*FOXM1*′s role is not restricted to humans; it has been identified and studied in a variety of species. In *Drosophila melanogaster*, the Forkhead transcription factor homologs are critical for wing and eye development, reflecting its broader role in orchestrating tissue-specific gene expression during development [11]. In mammals, particularly in *Mus musculus*, the *FOXM1* ortholog (*Foxm1*) has been shown to quickly switch on and off cell proliferation and differentiation pathways in several instances [12,13] using the intrinsically disordered nature of its protein structure, which can quickly switch from order to disorder and start/stop transcription [14].

In humans, the role of *FOXM1* in cancer is still debated. For example, in breast cancer, overexpression of *FOXM1* has been shown to be linked with tumor progression [15], while its downregulation is associated with an increase in metastasis [16]. The downstream effects of *FOXM1* in activating target genes are likely oncogenic, as *FOXM1* activates the transcription of several oncogenes, such as *MYCN* [17], *KRAS*, *MAFB*, and *NUP98* [18,19] (Table S1), on top of regulating key architectural mechanisms of the cell cycle, like mitotic spindle assembly and chromosome segregation, through its activation of genes like *AURKB*, *CCNB1* (Cyclin B1), and *PLK1* [20]. In addition to cell cycle control, *FOXM1* is involved in maintaining the DNA damage response by regulating the expression of genes such as *EXO1*, *XRCC1*, and *NBS1*, which are essential in DNA damage recognition and DNA repair via homologous recombination [21]. This dual role in promoting the cell cycle and preserving genomic stability makes *FOXM1* the final molecular judge of tumor initiation, as it controls both a source of mutations and the proliferative hallmark of cancer.

In cancer, *FOXM1* is generally considered a marker for poor prognosis in cancer survival studies [22], often as a co-effector with other cancer master regulators [23], such as *CENPF* in prostate cancer [24] and *MYB* in lymphoma [25]. However, while possessing a clear pro-proliferative nature, *FOXM1* is not considered a canonical oncogene, since it has not yet been causally linked to tumor initiation, and it is currently not included in the COSMIC Cancer Gene Census, also due to its lack of cancer-specific somatic mutations [18].

In this manuscript, we will describe the presence of *FOXM1* within the human proteome and across its vertebrate orthologs. We will then highlight the role of *FOXM1* as a consistent overexpressor in cancer across the vast catalogue of tumor molecular samples available in the TCGA database [26], by comparing cancer signals with the largest transcriptional database for healthy human tissues, GTEx [27]. Finally, we will investigate the presence of *FOXM1* in the tumor microenvironment via a relatively novel quantitative technology—single-cell RNA-Seq—by leveraging studies where cancer and non-cancer cells are identified and sequenced in the same samples. Our study should provide further evidence for the role of *FOXM1* as a co-occurring element of tumorigenesis.

## 2. Materials and Methods

### 2.1. Sequence Analysis

Human *FOXM1* isoforms were retrieved using NCBI RefSeq [28]. Domain identification for human *FOXM1* isoforms was performed using SMART [29] and the PFAM domain database [30]. *FOXM1* genomic alterations were calculated across TCGA [26] Pan-Cancer Atlas provided by cBio Portal, through their OncoPrint algorithm [31]. The position and frequency of somatic point mutations for *FOXM1* were calculated using COSMIC [32].

*FOXM1* putative orthologs in vertebrates were inferred using the NCBI orthology assignment annotation [33] (https://www.ncbi.nlm.nih.gov/gene/2305/ortholog, accessed on 25 November 2024), which reported, for each species, a single ortholog. We chose a single representative species for each of the following vertebrate subclades: birds (*Gallus gallus*), turtles (*Trachemys scripta elegans*), alligators (*Alligator sinensis*), lizards (*Elgaria multicarinata webbii*), amphibians (*Xenopus tropicalis*), coealacanths (*Latimeria chalumnae*), lungfishes (*Protopterus annectens*), bony fishes (*Danio rerio*), and cartilaginous fishes (*Callorhinchus milii*). For mammals, we picked one representative for monotremes (*Ornithoryncus anatinus*) and one for marsupials (*Gracilinanus agilis*). Amongst placental mammals, we picked one representative for rabbits (*Oryctolagus cuniculus*), two for rodents (*Mus musculus* and *Rattus norvegicus*), one for carnivores (*Canis lupus familiaris*), one for even-toed ungulates (*Capra hircus*), one for insectivores (*Erinaceus europaeus*), one for bats (*Rhinolophus ferrumequinum*), one for odd-toed ungulates (*Equus caballus*), one for pangolins (*Manis pentadactyla*), one for flying lemurs (*Cynocephalus volans*), one for tree shrews (*Tupaia chinensis*), one for afrotheria (*Trichechus manatus latirostris*), and one for armadillos (*Choloepus didactylus*). For primates, we picked three representatives: *Homo sapiens*, *Gorilla gorilla gorilla*, and *Pan troglodytes*. The sequence collection provided 31 distinct RefSeq entries. These 31 *FOXM1* orthologous protein sequences were aligned using the MUSCLE multiple sequence alignment algorithm [34]. The tree building step of the phylogenetic analysis for *FOXM1* was performed using the Neighbor-Joining method [35]. Branch confidence in the tree was calculated using bootstrapping with 100 replicates [36]. All the alignment, tree generation, and tree evaluation steps were performed using MEGA11 [37]. Graphical adjustments (color and branch thickness) to the tree were performed using Inkscape version 1.4 [38].

### 2.2. Bulk RNA-Seq

GTEx [27] and TCGA [26] data were preprocessed to remove batch effects and study-specific biases using the Wang method [39]. We used GTEx data as representative of normal tissues, and TCGA data (only tumor samples) as representative of tumor tissues. The following 15 TCGA cancers were selected based on the presence of a corresponding normal tissue dataset: BLCA (bladder cancer), BRCA (breast cancer), COAD (colon cancer), ESCA (esophageal cancer), KIRC (kidney clear cell cancer), KIRP (kidney papillary cell cancer), LIHC (liver hepatocellular cancer), LUAD (lung adenocarcinoma), LUSC (lung squamous cell carcinoma), PRAD (prostate adenocarcinoma), READ (rectal cancer), STAD (stomach adenocarcinoma), THCA (thyroid cancer), UCEC (uterine corpus endometrial cancer), and UCS (uterine carcinosarcoma). The following GTEx/TCGA pairing was applied to provide normal tissues to each of the tumor tissues: bladder/BLCA, breast/BRCA, colon/COAD, esophagus/ESCA, kidney/KIRC, kidney/KIRP, liver/LIHC, lung/LUAD, lung/LUSC, prostate/PRAD, colon/READ, stomach/STAD, thyroid/THCA, uterus/UCEC, and uterus/UCs.

All our analyses were performed on R version 4.4.1 [40]. Differential expression analysis for each tumor vs. normal contrast was performed using the *DESeq2* algorithm version 1.44 [41], which has been previously shown to be a robust choice for datasets of this size and context [42]. Survival analysis was performed using the R *survival* package version 3.6.4, and patient groups were divided into “*FOXM1* low” and “*FOXM1* high” using the median value of *FOXM1* expression as the breakpoint. Master regulator analysis of *FOXM1* transcriptional network was performed using the R *corto* package version 1.2.4 [43], for both the network generation and the enrichment tests, as described before [44]. Networks were generated with 200 bootstraps and *p*-value = 10^−10^ for individual edges, with the exception of the UCS dataset where the sample size was one order of magnitude lower than other datasets (*n* = 47), and the *p*-value was set to 10^−2^ to provide comparable networks with those of other datasets. All *p*-values were adjusted (padj) using the Benjamini–Hochberg method [45].

### 2.3. Single-Cell RNA-Seq

We obtained single-cell RNA-seq dataset for breast cancer [46], colorectal cancer [47], and lung cancer [48]. In order to load and process the data, we used Seurat version 5.0.1 [49] running on R version 4.4.1 [40]. All three datasets were log-normalized and scaled using Seurat NormalizeData() and ScaleData() functions with default parameters. The filtering process was double-checked using the original authors’ thresholds for detected raw gene counts, UMIs and fraction of mitochondrial reads, confirming that the downloaded datasets were pre-filtered as described in the original publications. In particular, for the breast cancer dataset, we removed cells with less than 200 genes, less than 250 UMI and cells with more than 20% of mitochondrial genes expressed. For the colorectal cancer dataset, we discarded cells if they had either less than 200 genes detected, less than 1000 reads, fewer than 500 UMIs, more than 50% of UMIs mapping mitochondrial genes, non-empty droplet false discovery rate less than 0.1 or if more than 5% of reads were estimated to be chimeric. Finally, for the lung cancer dataset, since the data object is integrated between several different studies, we used, as the authors intended, the less stringent thresholds on gene counts (<600), number of genes detected (<200) and mitochondrial gene expression (>20%).

We used Seurat’s RunPCA() function to calculate the first 30 principal components and used them to compute the UMAP coordinates via the RunUMAP() function. Preliminary data exploration on *FOXM1* expression across the three datasets was performed by extracting the log-normalized *FOXM1* expression data from the Seurat objects with the FetchData() function (layer = “data” parameter), distinguishing between control and cancer cells. Control and cancer *FOXM1* expression vectors for each cancer type were also used to perform one-sided non-parametric Wilcoxon tests in order to check whether *FOXM1* expression was higher in tumor cells. For distribution visualizations, we used the beeswarm R package version 0.4.0 [50].

## 3. Results

### 3.1. FOXM1 Sequence Across Species

**Figure 1:**
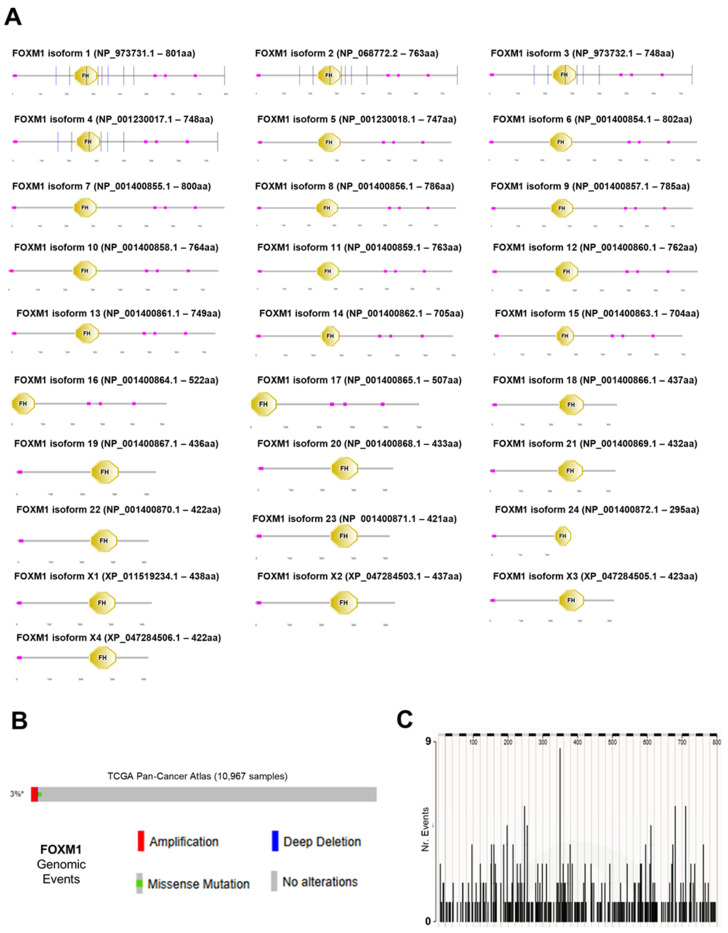
(**A**) Domain annotation of human *FOXM1* isoforms. The Forkhead (FH) domain is shown as an octagon. Purple boxes indicate low complexity regions. Vertical bars are provided by the SMART tool for the most studied isoforms and indicate the positions of intron/exon junction. (**B**) Genomic alteration detection for *FOXM1* across the TCGA Pan-Cancer Atlas (calculated by the cBioPortal OncoPrint algorithm). (**C**) Location of single-point somatic mutations in the *FOXM1* gene according to the COSMIC cancer catalogue.

**Figure 2:**
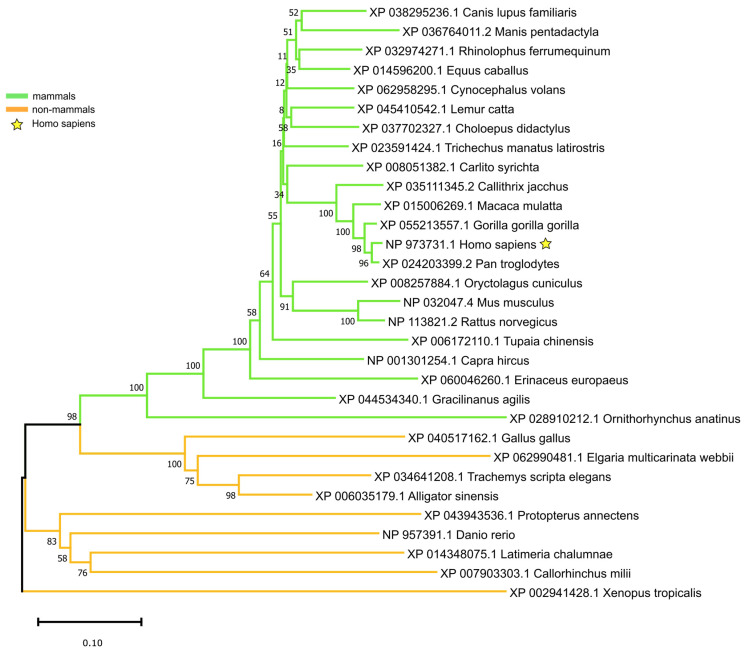
Phylogenetic tree of *FOXM1* orthologs in vertebrates. The optimal tree, calculated using the Neighbor-Joining method, is shown. 100 bootstrapping replicates were generated, and the percentage of replicate trees in which the downstream taxa grouped together is shown. The branch lengths are proportional to the number of amino acid substitutions per site, after the Poisson correction method was applied. Tree drawn with MEGA11 and Inkscape.

According to the most recent NCBI annotation, the human proteome contains 28 distinct *FOXM1* protein isoforms (24 RefSeq-validated and 4 predicted), ranging from 295 to 802 amino acids (Figure 1A). All human isoforms arise from alternative splicing, and all contain a complete Forkhead (FH) domain (Figure 1A). According to TCGA [26] and GTEx [27] data on physiological tissues, the most expressed *FOXM1* transcripts encode for isoforms 1 through 4, which are nearly identical in their sequence and account for >99.9% of the total expression of *FOXM1* [15]. Despite its role in cancer being acknowledged in literature [5,15], *FOXM1* is altered in only 3% of all human cancer genomes (collected in the TCGA Pan-Cancer Atlas), with amplifications being the most common event (Figure 1B), followed by somatic point mutations. According to the COSMIC catalogue for somatic cancer mutations [32], there is no focal point for *FOXM1* mutations, which are not only rare but also evenly distributed across its protein sequence (Figure 1C). We tested the presence of *FOXM1* across all vertebrate clades and generated an evolutionary model for this gene (Figure 2). *FOXM1* is present in all vertebrate clades as a single ortholog, according to the most recent orthology assignment pipelines by NCBI (Figure 2); we specifically searched for outparalogs of *FOXM1* [51] across the vertebrate group and, to the best of our knowledge, we found none, at least in the representative species of each vertebrate subclade.

### 3.2. FOXM1 Expression in Bulk Cancer RNA-Seq

**Figure 3:**
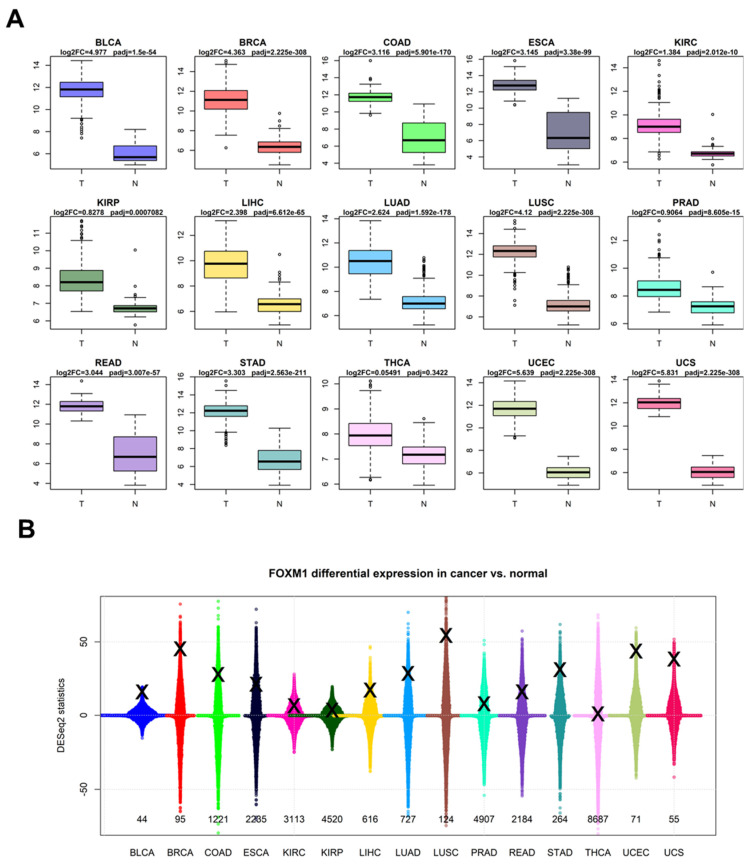
Expression of *FOXM1* across 15 human normal (GTEx) and cancer (TCGA) tissues. (**A**) Box plots indicting the difference between tumor (left) and normal (right) samples. (**B**) Beeswarm plots indicating gene-by-gene differential expression (expressed as DESeq2 negative binomial statistics) across 15 human cancer vs. normal comparisons. The X indicates the position of *FOXM1* in the gene ranking, and the number below each plot indicates the ranking of *FOXM1* in the upregulated transcriptome.

In our analysis, we investigated the presence of *FOXM1* transcripts in the two largest human bulk RNA-Seq datasets generated so far and publicly available: GTEx [27] and TCGA [26]. These collections of data report the transcriptome-wide expression of tens of thousands of patients, encompassing every human gene. We decided to design a comparative differential expression analysis by using the two datasets at the same time, after the appropriate batch correction (see Section 2). For each human cancer, we chose the histologically more appropriate normal tissue available on GTEx (see Section 2), in order to investigate whether the *FOXM1* transcript was altered in tumoral condition, relative to the most likely tissue of origin. We ultimately selected 15 cancer types with an appropriate comparative normal control and performed transcriptome-wide differential expression analysis. Our results strikingly show that *FOXM1* is upregulated in all the 15 tumor types, with a significant (*padj* < 0.01) adjusted *p*-value (derived from the DESeq2 negative binomial test) in 14 out of 15, the only exception being thyroid cancer, with *padj* = 0.3422 (Figure 3A). *FOXM1* is massively upregulated, with adjusted *p*-values below the limit of precision of the R software, so *p* < 2.2 × 10^−308^ in breast cancer (Log_2_ Fold Change = +4.36), lung squamous cancer (Log_2_ FC = +4.12), and the two uterine cancers (UCEC and UCS, with Log_2_ FC of +5.64 and +5.83). *FOXM1* is therefore upregulated in a pan-cancer range, across all TCGA datasets with a corresponding normal tissue in GTEx.

While the upregulation of *FOXM1* is a robust and monotonic result, and despite having corrected batch effects within and between datasets, one could argue that *FOXM1* is just upregulated in all tumors as a byproduct of the majority of the human transcriptome being upregulated in tumors. In order to test this, we calculated differential expression in the tumor vs. normal contrast for all human genes across the 15 tumor types (Table S2). We considered only coding genes and genes measured in all TCGA and GTEx datasets, amounting to a total of 20,242 genes, a number consistent with what is currently known on the total number of human coding genes [52]. This showed us that the transcriptional rewiring (and relative differential expression scores) induced by cancer affects genes in a symmetrical way, with equal numbers of up- and downregulated genes (Figure 3B). *FOXM1* is consistently upregulated, being in the top 150 of the most upregulated transcripts in some cancers (bladder, breast, lung squamous, and uterine cancers).

**Figure 4:**
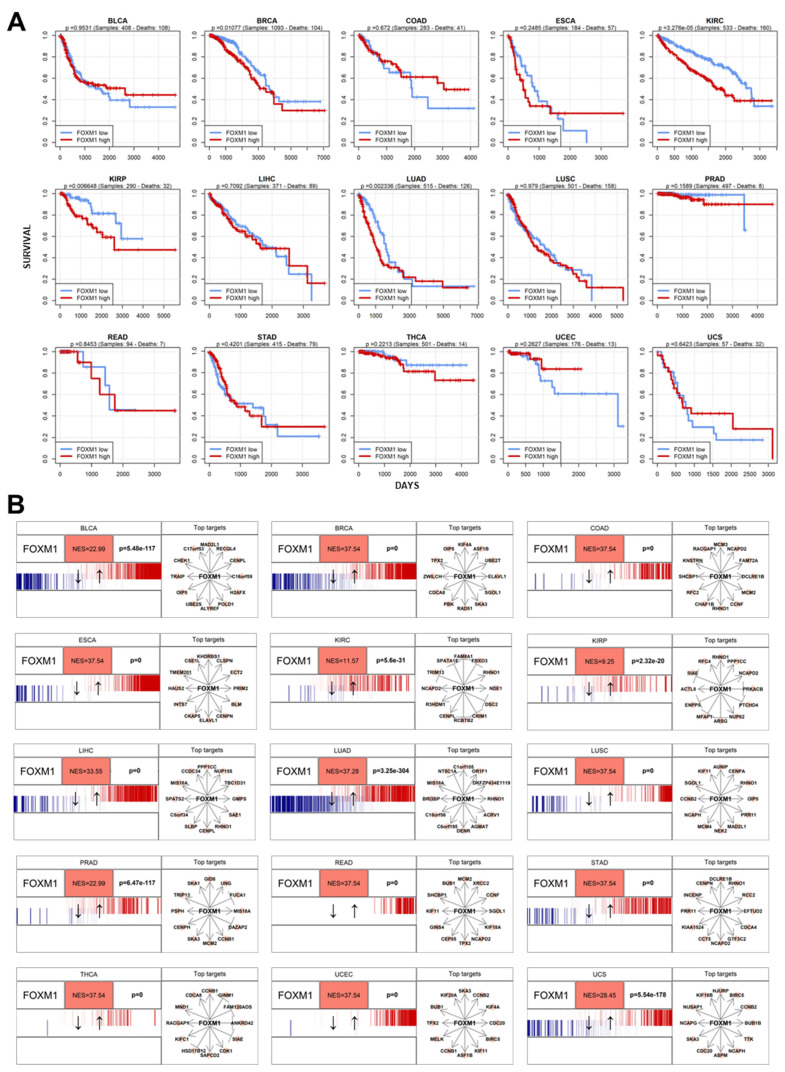
(**A**) Survival analysis of *FOXM1* for the 15 TCGA cancer types selected in this study. Patients were stratified in two groups: with *FOXM1* expression above the mean (“*FOXM1* high”, in red) and below the mean (“*FOXM1* low”, in blue). (**B**) Master regulator analysis of *FOXM1* across the 15 TCGA cancer types selected in this study.

While significantly upregulated in most cancer types, survival analysis does not indicate *FOXM1* as an immediate discriminant of mortality, as highlighted in Figure 4A. In fact, while a general pan-cancer tendency as a negative prognostic marker can be observed, survival of patients with a higher-than-median expression of *FOXM1* is significantly worse in four cancer types, specifically BRCA (*p* = 0.011), KIRC (*p* = 0.00023), KIRP (*p* = 0.0066), and LUAD (*p* = 0.0023).

The pan-cancer upregulation of *FOXM1* reverberates in its target genes, as highlighted by Master Regulator Analysis across the 15 selected cancer types. Collectively, the regulon of *FOXM1* is significantly upregulated across all selected cancer types, with no exception, with *p*-values close to or below the limit of R *p*-value precision (2.2 × 10^−308^) in BRCA, COAD, ESCA, LIHC, LUAD, LUSC, READ, STAD, THCA, and UCEC (Figure 4B).

### 3.3. FOXM1 Expression in the Cancer Microenvironment

Given the compelling expression patterns of *FOXM1* observed in bulk RNA-Seq data, we extended our analysis to scRNA-Seq datasets to determine whether similar upregulation patterns could be observed at the single-cell level. For this purpose, we utilized three publicly available scRNA-Seq atlases: the Breast Cancer Atlas (BrCA) from Wu et al. [46], the Colorectal Cancer Atlas (CRCA) from Pelka et al. [47], and the Lung Cancer Atlas (LuCA) from Salcher et al. [48]. To ensure accuracy, we performed standard preprocessing on each dataset (see Section 2). By leveraging the authors’ original metadata annotations, we meticulously excluded ambiguous and irrelevant cell populations, focusing exclusively on well-defined tumor cells and their matched healthy controls. This filtering step was essential to minimize background noise and enable precise comparisons of *FOXM1* expression levels across cancer and normal tissues.

**Figure 5:**
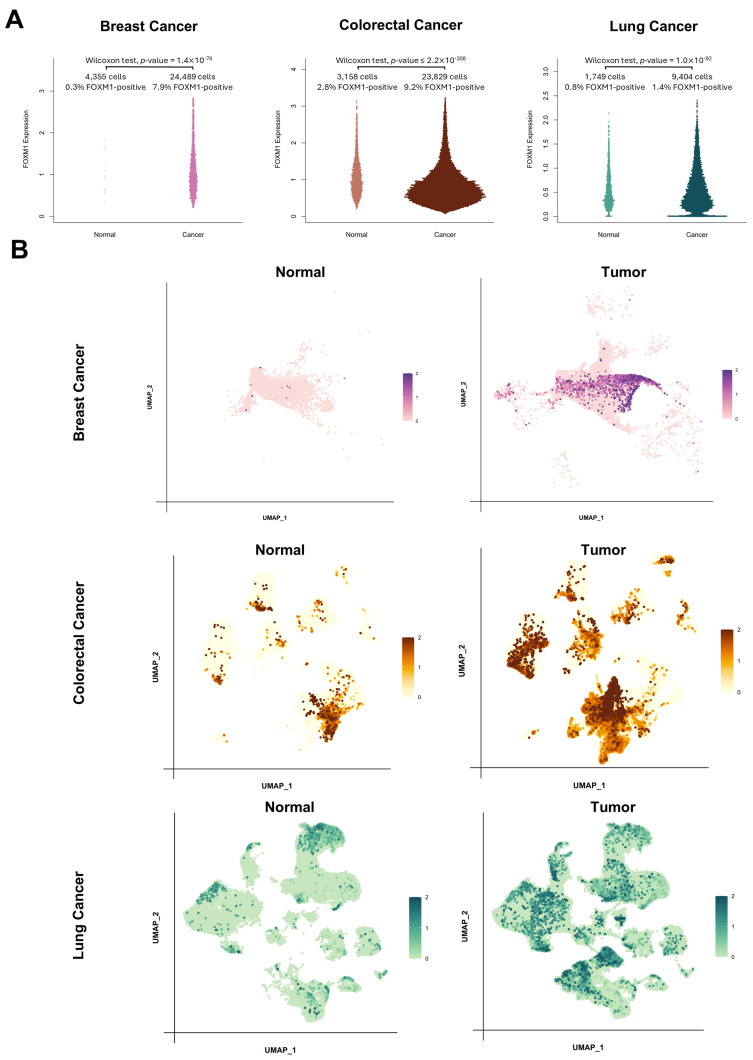
*FOXM1* in single cancer cells. (**A**) Beeswarm plots indicating the levels of *FOXM1* in normal cells (left) and tumor cells (right). (**B**) UMAP plots indicating the expression level of *FOXM1* (as LogScale-normalized FPKMs) in normal and tumor cells.

Our analysis revealed strikingly higher percentages of *FOXM1*-positive cells in the tumor groups compared to their normal counterparts (Figure 5A). We observed a significant and consistent upregulation of *FOXM1* in cancer cells, when compared to normal cells, across all three tumor microenvironments (*p* = 1.4 × 10^−76^ for BrCA, *p* = 1.0 ×10^−92^ for LuCA and *p* < 2.2 × 10^−308^ for CRCA. These findings further confirm the consistent upregulation of *FOXM1* not only in bulk but also in single-cell transcriptomics-wide data. Visualizing *FOXM1* expression using UMAP plots further corroborated these observations (Figure 5B): in the tumor datasets, we identified distinct clusters of cells exhibiting high *FOXM1* expression levels (Figure 5B). In contrast, healthy control datasets showed either no detectable *FOXM1* expression (BrCA) or only minimal expression levels (CRCA and LuCA). This pattern was consistent across all three types of cancer (Figure 5B).

Moreover, differential gene expression analysis (DGEA) provided additional evidence of *FOXM1* upregulation in cancer cells. *FOXM1* consistently ranked among the topmost highly expressed genes in the tumor datasets, with highly significant adjusted *p*-values below threshold levels. Specifically, *FOXM1* exhibited a Log_2_ FC of +1.22 with *p* < 2.2 × 10^−308^ (below R threshold) in CRCA, a Log_2_ FC of +0.94 with padj = 3.7 × 10^−88^ in LuCA, and a Log_2_ FC of +4.85 with padj = 7.6 × 10^−72^ in BrCA. These findings provide strong quantitative support for the role of *FOXM1* as a pan-cancer master regulator.

## 4. Discussion

Our analysis tested the *FOXM1* gene using the most recent sequence (and sequencing) data available at the time of writing (December 2024). According to our analysis, *FOXM1* is highly conserved and present in all vertebrates (Figure 2). The conservation of *FOXM1* is likely due to its critical role in cell cycle progression, cell differentiation, DNA damage repair and homeostasis [53], as has been observed for other genes with similar directional functions on core cellular mechanisms such as *DRG1* [54], *TCTP* [55], and *MCM4* [56]. Also, *FOXM1* appears as a single-copy transcription factor gene, with no detectable recent duplication event in vertebrates (Figure 2). *FOXM1* shares this low-duplicability feature with many cancer genes, which have been shown to possess a significantly lower chance to duplicate when compared to non-cancer genes [57]: the delicate pro-oncogenic nature of cancer genes likely poses a constraint to any change in their copy number and dosage, keeping cancer genes as evolutionary soloists.

We also tested *FOXM1* expression levels across 15 tumor types using the combination of the two largest human datasets for tumor (TCGA) and normal (GTEx) transcriptome-wide datasets, and detected a strikingly consistent upregulation of *FOXM1* in all tumor types (Figure 3). This upregulation is reflected also in the higher-resolution scenario of single-cell transcriptomics data, where *FOXM1* is upregulated in cancer cells within the same microenvironment in at least three tumor contexts (breast, colon and lung), both in absolute expression and number of cells with detectable *FOXM1* mRNAs (Figure 5).

While the upregulation of *FOXM1* in cancer contexts has been shown before [24], we present here that this gene is consistently upregulated in a pan-cancer manner, constituting a recurring mechanism cancer adopts to increase cell proliferation, one of the hallmarks of cancer [2]. In only one of the tumor types (THCA), *FOXM1* was not upregulated when compared to normal tissue (Figure 3A). While we have no final explanation for this finding, it can be stated that normal thyroid is already a proliferating tissue [58], which may mask the pro-proliferative effect of *FOXM1* in cancer. However, thanks to master regulator analysis, we at least observed that *FOXM1* transcriptional targets are upregulated in thyroid cancer when compared to normal thyroid tissue (Figure 4B).

Unlike canonical oncogenes, like *BRAF* [59] or *KRAS* [60], *FOXM1* is not commonly mutated nor amplified in cancer, warranting its exclusion from common “cancer gene lists” such as the Sanger Institute’s COSMIC Cancer Gene Census [18]. Despite this, *FOXM1* is one of the strongest pro-proliferative agents in human cancer and therefore constitutes an ideal candidate biomarker for tracking cancer progression; unlike some oncogenes or tumor suppressor genes, whose activation or inactivation is cancer-specific, activation of *FOXM1* is a consistent phenomenon spanning all neoplastic tissues. Being a transcription factor, the upregulation of *FOXM1* increasingly cascades into hundreds of transcriptional targets [61], further fueling its upregulation and pro-proliferative effects (Figure 4B). *FOXM1* is generally a negative predictor of survival (Figure 4A), with significantly negative prognostic power in four cancer types. In truth, other cancer types, such as PRAD and THCA, provide similar patterns but are limited by the low number of death events in the TCGA dataset (respectively, *n* = 8 and *n* = 14). Specifically for prostate cancer, when survival was tested using different datasets and more data, the negative prognostic power of *FOXM1* in cancer survival became apparent [24].

Given these properties, developing pharmacological solutions to inhibit *FOXM1* would seem like the perfect strategy to combat or mitigate tumor progression across all cancer types. However, three limitations to hypothetical anti-*FOXM1* pan-cancer therapy remain. The first is that correlation does not imply causation, especially in cancer [62], meaning that the upregulation of *FOXM1* could very well be a downstream pro-proliferative effect of a multi-directional upstream cancer-driving event; however, the direct causative role of *FOXM1* has been proven experimentally in at least one cancer type (PRAD) [24]. However, we do show in our analysis that *FOXM1* is likely one of the cancer master regulators (Figure 4B), which may further corroborate the notion of *FOXM1* as the ideal and central carrier of pro-proliferative signals. The second limitation is that *FOXM1*, like all human genes hijacked by cancer, also possesses a physiological pro-proliferative function, and it is also active in non-cancer cells. The blunt inhibition of *FOXM1*, without devices able to target it specifically towards cancer cells, would have detrimental effects on the overall health of the treated patient, much like standard untargeted chemotherapy [63]. The third and last limitation is that any anti-*FOXM1* therapy is impaired by the current lack of small molecules targeting this protein specifically, a problem shared by many non-hormone transcription factors [64]. If these limitations are overcome in the future, *FOXM1* could become the ideal focus not only for biomarker development but also for pan-anti-cancer therapy.

## 5. Conclusions 

The transcription factor *FOXM1* is conserved across vertebrates as a single-copy gene. Both its expression and the expression of its targets are significantly upregulated in 15 tumor types when compared to corresponding normal tissues. *FOXM1* is also upregulated in the tumor microenvironment, showing a marked upregulation in tumor cells compared to normal cells. *FOXM1* is, however, scarcely mutated in cancer and does not always strongly appear as a pan-cancer predictor for survival.

## Data Availability

No new raw data has been generated in this study.

## Supplementary Materials

The following supporting information can be downloaded at: https://www.mdpi.com/article/10.3390/genes16010056/s1, Table S1: intersection between *FOXM1* targets (defined in the Harmonizome catalogue) and oncogenes + tumor suppressor genes (defined in the Cancer Gene Census catalogue). Table S2: transcriptome-wide differential expression analysis for fifteen human tumor vs. normal contrasts.
